# The impact of COVID-19 on the tuberculosis control activities in Addis Ababa

**DOI:** 10.11604/pamj.2021.38.243.27132

**Published:** 2021-03-08

**Authors:** Negussie Wodajo Beyene, Alemu Lakew Sitotaw, Ben Tegegn, Kidist Bobosha

**Affiliations:** 1 Armauer Hansen Research Institute - Anti-Persoonsmijnen Ontmijnende Product Ontwikkeling (AHRI-APOPO) Tuberculosis Research Project, Armauer Hansen Research Institute, Addis Ababa, Ethiopia,; 2 Department of Biology, University of Antwerp, Antwerp, Belgium,; 3 Tuberculosis/Multi-Drug Resistance-Tuberculosis (TB/MDR-TB) Program, Addis Ababa City Administration Health Bureau, Addis Ababa, Ethiopia,; 4 Mycobacterium Diseases Research, Armauer Hansen Research Institute, Addis Ababa, Ethiopia

**Keywords:** COVID-19 control measures, tuberculosis control activities, Addis Ababa health centers

## Abstract

The first COVID-19 case was reported in Ethiopia on 13^th^ March 2020 and series of announcements of set of measures, proclamation and directives have been enacted to fight the coronavirus pandemic. These have implications for the regular health services including the TB control program. This brief communication assesses the impact of the COVID-19 response on the TB control activities of Addis Ababa health centers based on research project data. We compared the patient flows in pre-COVID-19 period (quarter 1, Q1) and during COVID-19 (quarter 2, Q2 and quarter 3, Q3) of 2020 at 56 health centers in Addis Ababa from all 10 sub-cities per sub-city. The patient flow declined from 3,473 in Q1 to 1,062 in Q2 and 1,074 in Q3, which is a decrease by 62-76% and 52-80% in Q2 and Q3 respectively as compared to that of Q1. In Q2, Kolfe keranio and Kirkos sub-cities recorded the biggest decline (76 and 75% respectively) whereas Yeka sub-city had the least decline (62%). In Q3, Kirkos sub-city had the biggest decline (80%) and Addis ketema sub-city had the lowest (52%). We conclude that the series of measures, state of emergency proclamation and government directives issued to counter the spread of COVID-19 and the public response to these significantly affected the TB control activities in Addis Ababa city as attested by the decrease in the patient flow at the clinics. Health authorities may inform the public that essential health services are still available and open to everyone in need of these services.

## Introduction

Ethiopia reported its first case of COVID-19 on 13^th^ March 2020 and on 16^th^ March 2020 the government suspended schools, large meetings, major sporting events, and university students were obliged to stay in campus while religious leaders were advised to reduce congregations for 15 days. On 8^th^ April 2020, state of emergency has been issued with more stringent prohibitions such as complete ban of any sort of meetings, handshake, serving alcohol at nightclubs and bars, public transports to serve with only 50% of capacity, etc. [1]. The public was in a panic-mode in the beginning and thus avoided going to public health facilities for fear of contracting COVID-19 [2]. This short communication assessed the impact of the government´s measures and the public response to it on the TB control activities of selected health centers in Addis Ababa using research project data.

## Methods

As part of the Armauer Hansen Research Institute, Anti-Persoonsmijnen Ontmijnende Product Ontwikkeling (AHRI-APOPO) tuberculosis research project, we have been collecting sputum samples from all consenting new presumptive pulmonary TB cases that provide sputum samples for Acid-Fast Bacilli (AFB) smear microscopic examination at 56 selected public health clinics in Addis Ababa from all 10 sub-cities. There were minimum of 4 and maximum of 8 participating health centers from each sub-city. There were 4 participating health centers from Guelele and Lideta sub-cities, 8 from Arada, 7 from Addis ketema and 3 sub-cities have 6 and the remaining 3 sub-cities have 5 participating health centers. Out of the 56 health centers, 3 were repurposed as COVID centers and thus excluded from further analysis. We investigated the change in patient flow (numbers of patients presenting with signs and symptoms for TB and meeting the criteria for TB testing) in the COVID period, Q2 and Q3 of 2020 as compared to that of the pre-COVID period, Q1 of 2020 at these clinics. Comparison in terms of percentage change in patient flow between the quarters was made at sub-city level rather than individual health centers.

## Results

**Figure 1:**
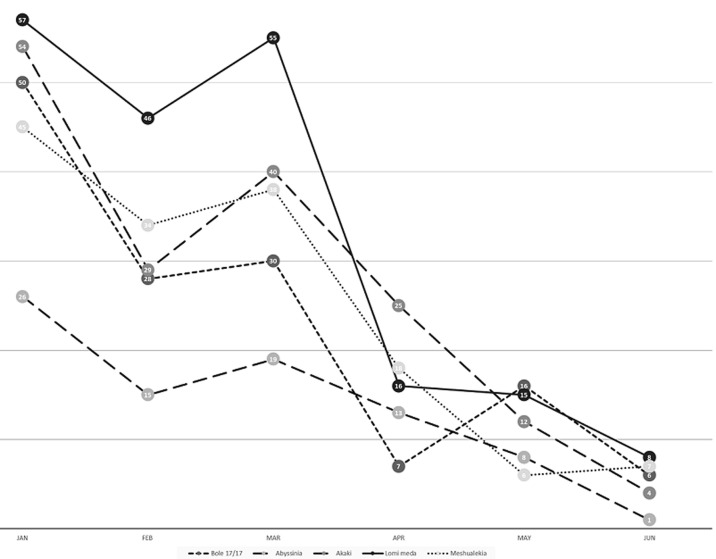
patient flow per month at 5 selected health centers in Q1 and Q2 of 2020

The number of patients with signs or symptoms of TB markedly decreased during the COVID-period in all sub-cities. Table 1 shows the patient flow in Q1, Q2 and Q3 for the health centers as per sub-cities. In Q2, the decline in the patient flow compared to Q1 was on average 69% and ranged from 62% in Yeka sub-city to 76% in Kolfe keranio sub-city. In Q3, the decline in patient flow compared to Q1 was on average 69% and ranged from 52% in Addis ketema sub-city to 80% in Kirkos sub-city. In both Q2 and Q3, Kirkos sub-city exhibited the biggest decline. The city-wide overall decline was also 69% in both Q2 and Q3. Figure 1 demonstrates the decline per month for 5 health centers selected based on the relatively large patient flow (Bole 17/17, Abyssinia from Addis ketema sub-city, Akaki from Akaki kaliti sub-city, Lomi meda from Kolfe keranio sub-city and Meshualekia from Kirkos sub-city), shedding additional light on how sharp the decline was in Q2.

**Table 1:** patient flow in quarter-1(Q1), quarter-2(Q2) and quarter 3(Q3) of 2020 at health centers from all 10 sub-cities

Sub-city	No of participating health centers	Number of patients presenting with signs and symptoms of TB and eligible for testing			% decline in Q2, (1-Q2/Q1)*100	% decline in Q3, (1-Q3/Q1)*100
		Q1	Q2	Q3		
Addis ketema	7	386	137	185	65	52
Akaki kaliti	5	327	117	108	64	67
Arada	8	326	80	143	75	56
Bole	6	433	150	115	65	73
Gulele	4	229	79	67	66	71
Kolfe keranio	6	534	130	128	76	76
Lideta	4	249	83	70	67	72
Nefasilk lafto	5	367	106	123	71	66
Kirkos	6	452	115	90	75	80
Yeka	5	170	65	45	62	74
Total	56	3473	1062	1074	69	69

## Discussion

Our analysis adds to the increasing body of evidence that the COVID-19 epidemic has a negative impact on TB control. The Stop TB Partnership Secretariat conducted a rapid assessment of the impact of COVID-19 pandemic response on the TB program activities of 20 high burden countries and found out that resources (staff, laboratory space, supplies, etc.) meant for the program have been shifted to the former in addition to mobility restrictions (lack of transport and lockdowns) that prevented patients to reach to the health facilities. They found an 80% decline in daily TB notification rate in India [3]. Another study from India reported a 70% decline in TB case notification in a 4-months period [4]. A similar comparison of presumptive TB cases at a Nigerian TB clinic for the period January to May 2019 and January to May 2020 revealed a decline by 35% in the latter period but a 90% decline for the months of April and May as compared to that of the same months in the previous year [5]. A 59% decline in Q2 as compared to Q1 was also reported in a small city in Eastern part of Ethiopia [6].

Our research data analysis shows a 69% decline in the number of presumptive TB patients eligible for testing in both Q2 and Q3. This has been acknowledged by the Deputy head of Addis Ababa City Administration Health Bureau in an interview with the 6^th^ May 2020 edition of Ethiopian Reporter (in Amharic) in which he pleaded to the public to continue seeking all types of health services at their nearby health facilities which are not designated for COVID-19 responses [7]. Note that out of the 56 health centers participating in our project, only 3 are selected as COVID centers which were excluded from this analysis. The decrease in presumptive pulmonary TB cases can likely be attributed not only to the government measures (restrictions on the capacity of transport services, physical distancing, etc.) that affects the health seeking behavior but also to the stigma associated with and fear of COVID-19. Stigmatization of TB is a known challenge especially in high burden sub-Saharan African countries where the prevalence of HIV/AIDS is also higher, and it´s sometimes perceived that having TB means the same as having HIV/AIDS [8]. Some of the signs and symptoms of TB and COVID-19 (cough, fever, sputum production, fatigue, breathlessness) are similar and those patients experiencing these symptoms may face double stigmatization for both TB and COVID-19. As a result, people may fear to present themselves to the health facility [9].

A modeling study based on the aforementioned assessment data collected from 20 high burden countries was commissioned by the Stop TB partnership in collaboration with the Imperial College London, Avenir Health and Johns Hopkins University to understand the long-term impact of COVID-19 on TB. The modelling revealed that a three-month lockdown and a 10-month restoration of full TB services would result in an additional 6.3 million TB cases and an additional 1.4 million TB deaths in the period 2020 to 2025 globally. That means in 2021, the global TB incidence and death might be thrown to the level we had five to eight years back discrediting all the global efforts made [10]. A similar study by the COVID-19 response team of Imperial College London predicted a 20% increase in TB deaths of high burden low- and middle-income countries in the next five post-pandemic years [11]. Both studies recommended continuation of TB diagnostic, treatment and prevention services during the pandemic and recovery periods. These recommendations have also been reiterated in the recent national guideline developed by the Ethiopian NTP that aims to ensure the continuity of TB care and preventive services in the era of COVID-19 pandemic and beyond.

## Conclusion

Our results suggest that the series of measures, state of emergency proclamation and government directives issued to counter the spread of COVID-19 and the public response to these markedly affected the TB control activities in Addis Ababa city. Despite the relaxation of some of the restrictions and the publics´ attitude of getting accustomed to living with COVID-19, still the patient flow was significantly lower in Q3. Therefore, the ministry of health and the city administration health bureau could make an impact by informing the public (through different media) that essential health services are still available and open to everyone in need of these services. Furthermore, the NTP in general and Addis Ababa City Administration Health Bureau in particular should consider the option of simultaneous screening of TB and COVID-19 and capitalize on the experience of TB clinic staff on contact tracing, mitigation of stigma, sample referral networks, and other related tasks [12]. The AHRI-APOPO, German leprosy and Tuberculosis Relief Association (GLRA) and the Federal Prison Administration team has recently started simultaneous screening of TB and COVID-19 in prison settings and is making good experiences in tackling the old and the new disease at the same time.

### What is known about this topic

- The COVID-19 pandemic affects health service delivery worldwide;
- TB service delivery significantly suffered in high TB burden countries but with limited quantitative data from sub-Saharan African countries.

### What this study adds

- Our study provides a quantitative evidence of the effect of COVID-19 on TB control program in Addis Ababa with a relatively large number of participating health centers;
- The results of this study showcase the impact of COVID-19 on TB control program of a high burden sub-Saharan African country which can be used by African and international actors supporting TB control endeavors in Africa.
